# A Finite State Machine Approach to Algorithmic Lateral Inhibition for Real-Time Motion Detection †

**DOI:** 10.3390/s18051420

**Published:** 2018-05-03

**Authors:** María T. López, Aurelio Bermúdez, Francisco Montero, José L. Sánchez, Antonio Fernández-Caballero

**Affiliations:** Departamento de Sistemas Informáticos, Universidad de Castilla-La Mancha, 02071-Albacete, Spain; maria.lbonal@uclm.es (M.T.L.); aurelio.bermudez@uclm.es (A.B.); francisco.msimarro@uclm.es (F.M.); jose.sgarcia@uclm.es (J.L.S.)

**Keywords:** formal model, finite state machines, artificial neural networks, motion detection, field programmable gate array

## Abstract

Many researchers have explored the relationship between recurrent neural networks and finite state machines. Finite state machines constitute the best-characterized computational model, whereas artificial neural networks have become a very successful tool for modeling and problem solving. The neurally-inspired lateral inhibition method, and its application to motion detection tasks, have been successfully implemented in recent years. In this paper, control knowledge of the algorithmic lateral inhibition (ALI) method is described and applied by means of finite state machines, in which the state space is constituted from the set of distinguishable cases of accumulated charge in a local memory. The article describes an ALI implementation for a motion detection task. For the implementation, we have chosen to use one of the members of the 16-nm Kintex UltraScale+ family of Xilinx FPGAs. FPGAs provide the necessary accuracy, resolution, and precision to run neural algorithms alongside current sensor technologies. The results offered in this paper demonstrate that this implementation provides accurate object tracking performance on several datasets, obtaining a high F-score value (0.86) for the most complex sequence used. Moreover, it outperforms implementations of a complete ALI algorithm and a simplified version of the ALI algorithm—named “accumulative computation”—which was run about ten years ago, now reaching real-time processing times that were simply not achievable at that time for ALI.

## 1. Introduction

Over recent decades, many researchers have explored the relationship between discrete-time recurrent neural networks and finite state machines, either by showing their computational equivalence or by training the former to perform as finite state recognizers [1]. The relationship between discrete-time recurrent neural networks and finite state machines has very deep roots [2,3]. Firstly, consider that finite state machines constitute the best-characterized computational model, whereas artificial neural networks have become a very successful tool for modeling and problem solving. Indeed, the fields of neural networks and finite state computation emerged simultaneously. A McCulloch-Pitts net really is a finite state machine of interconnected McCulloch-Pitts neurons, each of them in two possible states: firing and not firing [3]. Kleene formalized the sets of input sequences that led a McCulloch-Pitts network to a given state, and later, Minsky showed that any finite state machine can be simulated by a discrete-time recurrent neural net using McCulloch-Pitts units [2].

Let us also consider the fact that the use of neural networks for sequence processing tasks has a very important advantage: neural networks are trained to perform sequence processing tasks from examples. An important issue in the motivation of some of our papers is that the performance of neural networks—especially during learning phase—can be enhanced by encoding a priori knowledge about the problem directly into the networks [4,5]. This knowledge can be encoded into a recurrent neural network by means of finite state machines [6]. Indeed, sequence processors may be built around states. State-based sequence processors maintain and update, at each time step, a state that stores the information about the up-to-date input sequence, which is necessary to compute the current output [7]. The state is recursively computed from the state at previous times, and the current input using a suitable next-state function. The output is then computed using an output function.

Lateral inhibition (LI) is the basis of many discrete-time recurrent networks. Moreover, the lateral inhibition method and its application to motion detection tasks have been exploited in various computer vision applications [8]. A previous work by the same authors introduced a finite state machine version of the so-called algorithmic lateral inhibition (ALI) method, implemented in field programmable gate arrays (FPGAs) in order to detect moving objects in video [9]. Unfortunately, this method, fully inspired in LI, did not reach real-time and could not be applied to real-world applications. With the aim of attaining the necessary real-time performance, we used a model of the neutrally-inspired accumulative computation (AC) method, a simplified version of the ALI method in which the more time-consuming LI part of the algorithm was eliminated. AC was also implemented as a finite state machine in FPGAs [10].

Our persistent efforts through time in working with FPGAs is motivated by the continuous improvement of the performance of sensor technologies, which has triggered a clear increase in their use in new fields of application [11], with special emphasis on the computer vision area [12,13,14]. Indeed, FPGAs are flexible hardware platforms for developing accelerated implementations of artificial neural network models, providing high performance per watt of power consumption. In addition, continual growth in the size and functionality of FPGAs over recent years [15,16,17,18] has increased our interest in recalculating performance in FPGA-based real-time video processing over time. This is the reason why this paper revisits our previous FPGA-based ALI and AC implementations.

The main contributions of this paper are threefold. (1) The formal model of finite state machines that simplifies the general neurally-inspired ALI algorithm [19] is reproduced for ease of explanation; (2) The formal model is implemented in current Xilinx FPGAs to further speed up the processing time of the ALI algorithm; (3) A comparison between FPGA-based implementations of AC and ALI (about ten years ago) and ALI (to date) is made. In this way, we will establish a relationship between technological advancements and the possibility of facing more complex and accurate motion-detection algorithms.

The remainder of this paper is as follows. Section 2 describes the neurally-inspired lateral inhibition method as a biological precursor for the computation of the ALI method. Section 3 revisits the ALI algorithms for the motion-detection task. Afterwards, Section 4 introduces the hardware implementation of ALI for motion-detection in current FPGAs. Section 5 describes some results obtained on several datasets. Lastly, Section 6 presents the main conclusions reached in this study.

## 2. The Algorithmic Lateral Inhibition Method

Computational neuroscience is characterized by the desire to fulfill two clear objectives [20], namely: (1) the construction of computational models of neurons and neural networks as a valuable tool to understand the nervous system; and (2) the use of neural models of biological inspiration as methods for solving problems in a wide range of domains, such as vision, character recognition, temporal series prediction, planning, and control, where symbolic methods have shown to be inadequate or insufficient.

We use biology as a source of inspiration to obtain methods and procedures useful in engineering and computation. Concretely, we look for inspiration in neural networks which repeat along the whole visual pathway for artificial vision and motion related problems. If we had to take a neural circuit that insistently repeats itself in the superior vertebrates’ visual path in looking for inspiration for the development of artificial neural networks that may be useful in computer vision, there is no doubt that such a circuit would be of lateral inhibition (LI). LI inhibition refers to the inhibition effect that neighboring neurons in brain pathways have upon each other. More precisely, LI is the capacity of an excited neuron to reduce the activity of its neighbors. Such a structure of neural calculation, in its non-recurrent (guided by data), as well as in its recurrent (guided by local results) versions, appears along the whole visual pathway. Firstly, it appears among cones and rods in bipolar, amacrine and ganglion cells. It also appears in the lateral geniculate body, and finally, among columns in the cerebral cortex. Thus, it is reasonable to think of the very special value of LIs in the process of constructing an internal representation of a visual scene [21].

In the field of neural computation, LIs have essentially been used in two kinds of tasks. They have been used as filtering tasks to detect spatial-temporal contrasts, as well as in preprocessing tasks in learning networks. In the latter, before beginning to modify the weight values, the “winner neuron” is selected as the one that responds with greater intensity to a given configuration of stimuli. This is performed by soft-competition methods, with much accentuated nucleus in differences, or by hard-competition methods (“winner take all”, WTA) [22]. In a linear formulation of the LI, a convolution operator with a nucleus in differences is used in such a way that the geometry of the nucleus (symmetry, orientation, etc.) defines an important part of the calculus. Thus, in this same sense of recursive or non-recursive linear filters, the LI is also used in digital image processing to detect spatial, temporal or spatial-temporal contrasts.

Our proposal to increase the calculation capacity of lateral inhibition circuits is to maintain the relational structure, that is to say, to maintain the skeletal model of the LI, but to substitute the usual analytic operators in the linear models by others which are logical-relational in nature, of a greater calculation capacity [23,24]. In this way, the concept of lateral inhibition is extended to embrace a wider group of operators than the lineal and non-lineal analytic ones. In a computational sense, we are speaking of lateral inhibition algorithms, with non-linearity of “if-then” type, local memory and sequential control.

Finally, one more step in the generalization of the LI mechanism is to abstract the structure that underlies the anatomic circuits of the superior vertebrates’ visual path up to the knowledge level. The LI turns into a procedure to break up the subtasks where expressions are evaluated in the central part, the same or other expressions are evaluated over the data of the periphery, and there is a “dialogue” comparing the results of both evaluations of the central and the peripheral part [19]. In this work (and some previous ones) we use an abstract representation of the LI anatomic-physiological processes to build a method of an inferential nature at the knowledge level, and to test its usefulness within the context of computer vision. Algorithmic lateral inhibition (ALI) is the symbolic/inferential version of LI in which analytical operators are replaced by rules [24].

In this work, we show some temporal non-recurrent and spatial recurrent ALI processes, as described in the previously referenced work [24]. We look at the results of accumulations in the central and peripheral parts, and the later competitions (usually recursively calculating a consensus value between central and peripheral accumulations), and its usefulness in the construction of an internal representation of the moving pixels that are present in a video sequence. From now on, this paper will focus on the formal model for the ALI applied to motion detection in video sequences. The article shows how to implement ALI in motion detection by means of a formal model described as finite state machines. These are concretely called *ALI Temporal Motion Detecting, ALI Spatial-Temporal Recharging* and *ALI Spatial-Temporal Homogenization*.

## 3. Formal Model of ALI for Motion Detection

The control knowledge of the ALI method is described extensively in Section 3.1,Section 3.2 and Section 3.3 by means of finite state machines in which the state space is constituted from the set of distinguishable cases in the state of accumulated charge in a local memory [10]. The general ALI method can be broadly described as follows:

- A scalar quantization into N levels of accumulated charges is performed on the input images.
- For each level, if an image pixel at time t does not belong to the level, the charge at that pixel and that level is discharged down to minimum value $v_{dis}$.
- For each level, if an image pixel at time t belongs to the level and did not belong to it at previous time t−Δt, the charge value is loaded to the maximum saturation value $v_{sat}$.
- For each level, if an image pixel belongs to the level at time t and t−Δt, the charge is decremented by value $v_{dm}$ (discharge value due to motion detection). Of course, the charge value cannot be under minimum value $v_{dis}$. The discharge of a pixel by quantity $v_{dm}\;$ is the way to stop paying attention to a pixel of the image through time.
- For each level, a pixel not directly or indirectly linked by means of lateral inhibition mechanisms to a maximally charged pixel ($v_{sat}$), decreases to total discharge $v_{dis}\;$ with time. Therefore, an extra charge $v_{rv}$ (charge value due to neighborhood) is added to the charge in those image pixels that receive a recharge stimulus from any of the four neighboring pixels.
- Lastly, the charge values from all levels are fused into a single output image, where moving objects are given a homogeneous charge value through a set of recurrent lateral inhibition processes among all neighbors that possess a certain minimum charge value.

Thus, we distinguish N states, $S_{0},S_{1},\ldots ,S_{N-1}$, where $S_{0}$ is the state corresponding to the totally discharged local memory ($v_{dis}$; in general, $v_{dis}=0$), $S_{N-1}$ is the state of complete charge ($v_{sat}$; in general, $v_{sat}=255$), and the rest are the (N−2) intermediate charge states between $v_{dis}$ and $v_{sat}$.

Let us suppose, without loss of generality, that it is enough to distinguish eight levels of accumulated charge (N=8). Consequently, we can use an 8-state automaton ($S_{0},S_{1},\ldots ,S_{7}$), where $S_{0}$ corresponds to $v_{dis}$ and $S_{7}$ to $v_{sat}$, as a formal model describing the data flow corresponding to the calculation of the subtasks. Let us also suppose that discharge and recharge initially take the values corresponding to the descent of two states ($v_{dm}=64$), and to the ascent of one state ($v_{rv}=32$). This way, the state transition diagram corresponds to a kind of reversible counter (“up-down”) which is controlled by the result of lateral inhibition (dialogue among neighbors).

To complete the description of the states, together with the accumulated charge value, *v* ($v_{dis}\leq v\leq v_{sat}$), it is necessary to include a binary variable, $A_{C}=\left\{0,\;1\right\}$. When $A_{C}=1$, a pixel tells its neighbors that it has detected a moving object, or that some neighbor has told it to have detected such moving object. This is the label that informs the presence of a moving object in the receptive field (in the central part or in the periphery). Thus, state S(t) is a tuple $S\left(t\right)=\left[v\left(t\right),\;A_{C}\left(t\right)\right]$.

**Figure 1:**
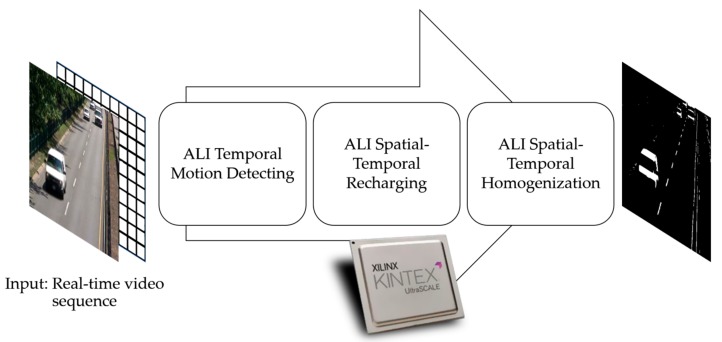
Deployment of the ALI method for use in motion detection.

Figure 1 anticipates the different phases of the ALI algorithm applied to motion detection. The three phases, to which the sequence of input video images is subjected, are explained in detail in the following sections. Note that *ALI Spatial-Temporal Homogenization* is the single phase with a clear lateral inhibition inspiration, which makes it a far more computationally expensive one. The simplification denominated accumulative computation (AC) covers only the first and second phases.

### 3.1. ALI Temporal Motion Detecting

The aim of this phase is to detect the temporal and local (pixel to pixel) contrasts of pairs of consecutive binarized images at gray level k. The phase firstly gets the values of the L=256 gray level input pixels I(i,j;t) as input data, and generates N=8 binary images, $x_{k}\left(i,j;t\right)$, corresponding to N levels obtained through scalar quantization. The output space has a memory with two levels: one for the current value, the other for the value of the previous instant. Thus, for N levels, there are 2N=16 binary values for each input pixel; at each level, there is the current value $x_{k}\left(i,j;t\right)$ and the previous value $x_{k}\left(i,j;t-\Delta t\right)$, such that:

(1) $$\begin{array}{cc} x_{k}\left(i,j;t\right)= & \{\begin{array}{ll} 1, & \mathrm{if}\;I\left(i,j;t\right)\in \left[\frac{L}{N}\cdot k,\;\frac{L}{N}\cdot \left(k+1\right)-1\right]\\ 0, & \mathrm{otherwise} \end{array} \end{array}$$

where k=0,…,N−1, is the level index. Thus, the first step is a scalar quantization algorithm called multilevel thresholding, that segments the image into *N* equally spaced gray levels.

A pair of binarized values at each level, $x_{k}\left(i,j;t\right)$ and $x_{k}\left(i,j;t-\Delta t\right)$, constitutes the input space to the temporal non-recurrent ALI. The output space is the result of the individual calculation phase in each element and the current charge value that initially is $v_{dis}$ at state $S_{0}$. It is formed by potential values $v_{dis},v_{sat}\;$and$\;\max\left\{y_{k}\left(i,j;t-\Delta t\right)-v_{dm},v_{dis}\right\}$, where $v_{dm}$ is the decrement value, $v_{dis}$ is the minimum charge value and $v_{sat}$ is the maximum charge value. Value $v_{sat}$ is obtained either when an object just enters the receptive field, or when movement has been detected by any of the pixel’s neighbors.

Thus, the output of phase *ALI Temporal Motion Detecting* is the accumulated charge value, $y_{k}\left(i,j;t\right)$, in association with label $A_{C}$. Remember that $A_{C}=1$ denotes the fact that a movement has been locally detected by this pixel.

(2) $$\begin{array}{cc} A_{C}= & \{\begin{array}{ll} 1, & \mathrm{if}\;(x_{k}\left(i,j;t\right)=1)\cap \left(x_{k}\left(i,j;t-\Delta t\right)=0\right)\\ 0, & \mathrm{otherwise} \end{array} \end{array}$$

(3) $$\begin{array}{cc} y_{k}\left(i,j;t\right)= & \{\begin{array}{ll} v_{sat}, & \mathrm{if}\;(x_{k}\left(i,j;t\right)=1)\cap \left(x_{k}\left(i,j;t-\Delta t\right)=0\right)\\ \max\left\{y_{k}\left(i,j;t-\Delta t\right)-v_{dm},v_{dis}\right\}, & \mathrm{if}\;(x_{k}\left(i,j;t\right)=1)\cap \left(x_{k}\left(i,j;t-\Delta t\right)=1\right)\\ v_{dis}, & \mathrm{otherwise}\; \end{array} \end{array}$$

**Figure 2:**
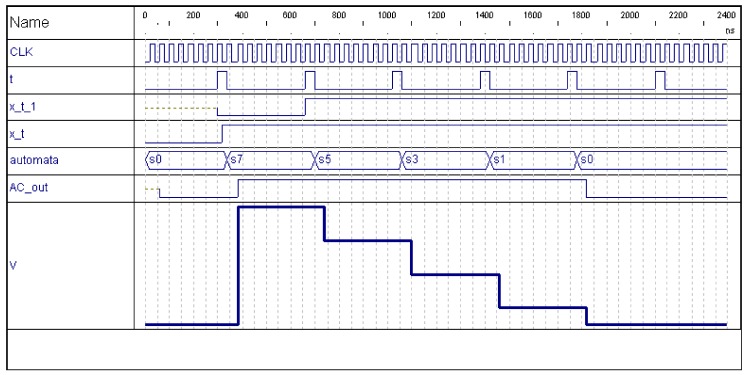
Evolution of the automata on an isolated image pixel (8 states).

The following transitions can be observed:

1. $x_{k}\left(i,j;t-\Delta t\right)=\left\{0,1\right\},\;x_{k}\left(i,j;t\right)=0$. In this case the calculation element (i,j) has not detected any contrast with respect to the input of a moving object in that level ($x_{k}\left(i,j;t\right)=0$). It may have detected it (or not) in the previous interval ($x_{k}\left(i,j;t-\Delta t\right)=1,\;x_{k}\left(i,j;t\right)=0$). In any case, the element passes to state $S_{0}\left[v=v_{dis},\;A_{C}=0\right]$, the state of complete discharge, independently of the initial state.
2. $x_{k}\left(i,j;t-\Delta t\right)=0,\;x_{k}\left(i,j;t\right)=1$. The calculation element has detected a contrast in its level ($x_{k}\left(i,j;t\right)=1$) in t, and it did not in the previous interval ($x_{k}\left(i,j;t-\Delta t\right)=0$). It passes to state $S_{7}\left[v=v_{sat},\;A_{C}=1\right]$, the state of total charge, independently of the previous state. Also, $A_{C}\;$passes to 1, to tell its potential dialogue neighbors that this pixel has detected a moving object. This fact will be used later during phase *ALI Spatial-Temporal Recharging*. Figure 2 shows, in first place (300 to 400 ns), the evolution of the automata states when motion is detected in a pixel where previously no motion was detected ($x_{k}\left(i,j;t-\Delta t\right)=0,\;x_{k}\left(i,j;t\right)=1$), passing from $S_{0}\left[v=v_{dis},\;A_{C}=0\right]$ to $S_{7}\left[v=v_{sat},\;A_{C}=1\right]$. Notice that CLK and t show the Δt and t time clock intervals, respectively. V and AC_out represent v and $A_{C}$.
3. $x_{k}\left(i,j;t-\Delta t\right)=1,\;x_{k}\left(i,j;t\right)=1$. The calculation element has detected the presence of an object in its level ($x_{k}\left(i,j;t\right)=1$), and it also detected it in the previous interval ($x_{k}\left(i,j;t-\Delta t\right)=1$). In this case, it diminishes its charge value by $v_{dm}$, corresponding to two states. This partial discharge can proceed from an initial state of saturation $S_{7}\left[v=v_{sat},\;A_{C}=1\right]$, or from some intermediate state ($S_{6},\;\ldots ,\;S_{1}$). This partial discharge due to the persistence of the object in that position and in that level is described by means of a transition from $S_{7}$ to an intermediate state, $S_{int}\left[v=v_{int},\;A_{C}=\left\{0,1\right\}\right]$, without going below the fully discharged level, $S_{0}\left[v=v_{dis},\;A_{C}=0\right]$. The descent in the element’s state is equivalent to the descent in the pixel’s charge, such that (as you may appreciate in Figure 2, starting around 670 ns) only the following transitions are allowed: $S_{7}⟶S_{5},\;S_{6}⟶S_{4},\;S_{5}⟶S_{3},\;S_{4}⟶S_{2},\;S_{3}⟶S_{1},\;S_{2}⟶S_{0},$ and $S_{1}⟶S_{0}$.

### 3.2. ALI Spatial-Temporal Recharging

In the previous phase, *ALI Temporal Motion Detecting*, we have obtained the individual “opinion” of each computation element. However, our aim is also to consider the “opinions” of the neighbors. The reason is that an individual element should stop paying attention to motion detected in the past, but before making that decision, there should be a communication in form of lateral inhibition with its neighbors, to see if any of them are in state $S_{7}$ ($v_{sat}$, maximum charge). Otherwise, it will be discharging down to $S_{0}\;$($v_{dis}$, minimum charge), because that pixel is not bound to a pixel that has detected motion. In other words, the aim of this phase is to focus on those pixels charged with an intermediate accumulated charge value, but directly or indirectly connected to saturated pixels (in state $S_{7}$) by incrementing their charge.

These “motion values” of the previous layer constitute the input space, whereas the output is formed by charge value $z_{k}\left(i,j;t\right)\;$after dialogue with neighboring pixels. The values of accumulated charge before dialogue are written in the central part of the output space of each pixel that now enters the dialogue phase according to the recurrent ALI scheme instantiated for this task. The data in the periphery of receptive field in the output space of each pixel now contains the individual calculations of its neighbors.

Let $v_{C}\left(t\right)=y_{k}\left(i,j;t\right)$ be the initial charge value at this phase. Each pixel considers the set of individual calculations, $\left\{v_{C}\left(t+l\cdot \Delta \tau \right),A_{j}\right\}$, by means of a logical union of labels $A_{j}$:

(4) $$A_{P*}\left(\tau \right)=\underset{j}{\cup }A_{j}\left(\tau \right)$$

This result, $A_{P*}$, is now used to output the new consensus charge value after dialogue, $z_{k}\left(i,j;t+\Delta t\right)$, with Δt=m·Δτ, being m≥l the number of iterations in the dialogue phase, a function of the size of the receptive field. The whole dialogue process is executed with clock τ, during m intervals Δ*τ*. It starts when clock t detects the configuration $x_{k}\left(i,j;t-\Delta t\right)=x_{k}\left(i,j;t\right)=1$ and terminates at the end of Δt, when a new image is considered.

(5) $$A_{C}=\{\begin{array}{ll} 1, & \mathrm{if}\;\left(v_{C}\left(t+m\cdot \Delta \tau \right)=v_{sat}\right)\cup \left(\left(v_{dis}<v_{C}\left(t+m\cdot \Delta \tau \right)<v_{sat}\right)\cap \left(A_{P*}=1\right)\right)\\ 0, & \mathrm{otherwise} \end{array}$$

(6) $$\begin{array}{cc} v\left(t+m\cdot \Delta \tau \right)= & \{\begin{array}{ll} v_{dis}, & \mathrm{if}\;v_{C}\left(t+m\cdot \Delta \tau \right)=v_{dis}\\ v_{sat}, & \mathrm{if}\;v_{C}\left(t+m\cdot \Delta \tau \right)=v_{sat}\\ \min\left\{v\left(t+m\cdot \Delta \tau \right)+v_{rv},v_{sat}\right\}, & \mathrm{if}\;(v_{dis}<v_{C}\left(t+m\cdot \Delta \tau \right)<v_{sat})\cap \left(A_{P*}=1\right) \end{array} \end{array}$$

At each dialogue step (in other words, at each interval of clock Δτ), the calculation element only considers values $x_{k}\left(i,j;t-\Delta t\right),\;x_{k}\left(i,j;t\right)$ and $A_{C}$ present in that moment in its receptive field. To diffuse or to use more distant information, new dialogue steps are necessary. In other words, new inhibitions in l·Δτ (1<l≤m) are required. This only affects state variable $A_{C}\left(\tau \right)$, as $x_{k}\left(i,j;t-\Delta t\right)\;$and $x_{k}\left(i,j;t\right)\;$values remain constant during the intervals used to diffuse τ and reach consensus on the different partial results obtained by the calculation elements.

Note that the recharge may only be performed once during all the dialogue steps. That is why $A_{C}=0$ when a recharge takes place. Lastly, the output will be:

(7) $$z_{k}\left(i,j;t+\Delta t\right)=v_{C}\left(t+\Delta t\right)$$

**Figure 3:**
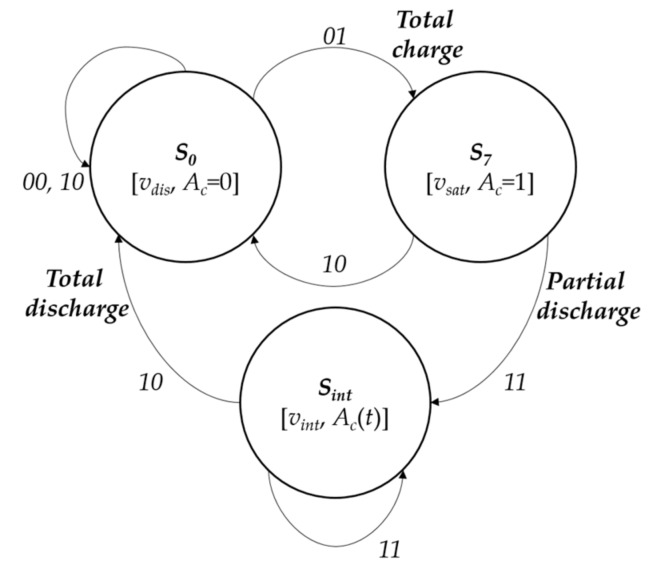
Control automaton that receives inputs $x_{k}\left(i,j;t-\Delta t\right)$ and $x_{k}\left(i,j;t\right)$, and produces three outputs, coincident with its three distinguishable charge states ($S_{0}=v_{dis},\;S_{7}=v_{sat}$, and {$v_{int}$}).

**Figure 4:**
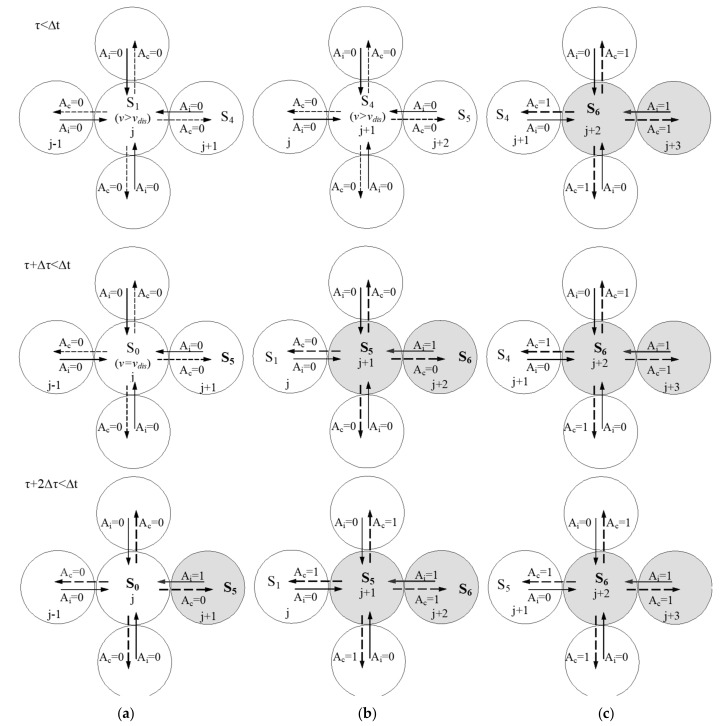
Detail of the dialogue where diffusion of motion detection is shown through “transparent” pixels (*j +* 2 and *j +* 1), while pixel *j* deserves an “opaque” behavior. Dialogue at (**a**) pixel *j*, (**b**) pixel *j +* 1, and (**c**) pixel *j +* 2, respectively.

Figure 3 shows the simplified state transition diagram, where the following transitions are distinguished:

1. $x_{k}\left(i,j;t-\Delta t\right)=\left\{0,1\right\},\;x_{k}\left(i,j;t\right)=0$. In any case, independently of the pixel’s dialogue with the neighbors (see Figure 4), at the end of Δt the pixel passes to state $S_{0}\left[v=v_{dis},\;A_{c}=0\right]$.
2. $x_{k}\left(i,j;t-\Delta t\right)=0,\;x_{k}\left(i,j;t\right)=1.\;$Again, independently of the dialogue step, the pixel’s state will be $S_{7}\left[v=v_{sat},\;A_{C}=1\right]$.
3. $x_{k}\left(i,j;t-\Delta t\right)=1,\;x_{k}\left(i,j;t\right)=1$.
  - **Local memory is in**$S_{0}\left[v=v_{dis},\;A_{C}=0\right]$. Pixels in state $S_{0}\;$are not affected by recharge due to motion detection in their periphery. Thus, the pixel maintains the same state *S_0_*.
  - **Local memory is in**$S_{7}\left[v=v_{sat},\;A_{C}=1\right]$. Pixels in state $S_{7}$ are maximally charged. Therefore, they cannot be recharged. They also remain in the same state.
  - **Local memory is in**$S_{int}\left[v=v_{int},\;A_{C}\left(\tau \right)\right].$ Depending on their four neighbors’ charge values, it can stay in $S_{int}$ if all neighbors have variable $A_{j}=0$, or transit up to $S_{7}$ if it finds some neighbor with variable $A_{j}=1$.
    - **Transit from**$S_{i}$** to **$S_{i+1}$. After recharge, the calculation element is now in $S_{i+1}.$ It sends $A_{C}=1$ and waits to the end of Δt. In a second clock cycle, Δτ, $A_{C}=1$ is potentially used by its neighbors to increment their charge values. Thus, the dialogue extends in steps of size the receptive field. Pixels with $A_{C}=1$ are said to be “transparent” if they allow information on motion detection by some neighbor (in state $S_{7}$) of their receptive field to cross them.
    - **Remain in **$S_{i}$**_. _**If none of its neighbors has transmitted $A_{j}=1$, the pixel stays in $S_{i}$, without recharging in the first Δτ. In this case, it maintains its proper $A_{C*}=0$, and its behavior is called “opaque”. However, if in a later Δτ and inside the dialogue interval it does receive any $A_{j}=1,$ it will pass to $S_{i+1}.$ Figure 4 illustrates this diffusion mechanism through “opaque” and “transparent” pixels of the receptive field.

Moreover, Figure 4 offers, in more detail, an example of a dialogue among j, j+1 and j+2. Pixels j+1 and j+2 are neighbors of pixel j. More concretely, Figure 4 shows the automata’s evolution when there is motion in both neighboring pixels.

### 3.3. ALI Spatial-Temporal Homogenization

The aim of this third phase is to obtain all moving patches present in the scene. The phase considers the union of pixels that are physically together and at a same gray level to be a component of an object. A set of recurrent lateral inhibition processes are performed to distribute the charge among all neighbors that are not fully discharged ($z_{k}\left(i,j;t\right)$ of the previous phase); those pixels are in states $S_{1}$ to $S_{7},$ and are physically connected. A double objective is pursued:

1. To dilute the charge due to the image background motion among other pixels of their own background, so that only moving objects are detected. To dilute the charge due to the image background motion does not mean that we are dealing with moving cameras. Instead, we are facing the problem of false motion detected where moving objects are just leaving pixels that now belong to the background.
2. To obtain a parameter common to all pixels of the object, those belonging to the same gray level (simple classification task).

Charge values, $z_{k}\left(i,j;t+\Delta t\right)$, offered by the previous phase, are now evaluated in the center and in the periphery. Now, let $v\left(t+\Delta t\right)=z_{k}\left(i,j;t+\Delta t\right)$ be the initial charge value at this phase. In this last phase, we have the average of those neighbors that hold charge values greater than a threshold value $\theta_{min}$.

(8) $$v_{C}=\max\{v_{C},{\;\theta }_{min}\}$$

We compare the result of the individual value in the center (C) with the mean value in the periphery (P), and produce a discrepancy class according to threshold, $\theta_{min}$, and pass the mean charge values that overcome that threshold. After this, the result is again compared with a second threshold, namely $\theta_{max}$, eliminating noisy pixels pertaining to non-moving objects.

(9) $$\begin{array}{cc} O_{k}\left(i,j;t+\Delta t\right)\;= & \{\begin{array}{ll} \theta_{min}, & \mathrm{if}\;v_{C}={\;\theta }_{min}\\ \frac{v_{C}+v_{P}}{2}, & \mathrm{if}\;\left(\theta_{min}<v_{C}<v_{sat}\right)\cap \left(\theta_{min}<v_{P}<v_{sat}\right)\\ v_{C}, & \mathrm{if}\;(\theta_{min}<v_{C}<v_{sat})\cap \left(v_{P}=\theta_{min}\right) \end{array} \end{array}$$

(10) $$O_{k}\left(i,j;t+\Delta t\right)=v_{dis},\;\;\;\mathrm{if}\;O_{k}\left(i,j;t+\Delta t\right)>\;\theta_{max}$$

The dialogue scheme and the description of the control automaton, where the transitions between the initial state $S_{i}\left(t\right)$ and the final state $S_{i}\left(t+\Delta t\right)$ state, are carried out, in agreement with rule:

(11) $$S_{i\;\mathrm{final}}=\frac{1}{N_{k+1}}\left(S_{i\;\mathrm{initial}}+\underset{j\in {\mathrm{RF}}_{k}}{\sum }v_{j}\right)$$

where the sum on sub-index *j* extends to all neighbors, $v_{j}$, belonging to the subset of the two-dimensional receptive field, ${\mathrm{RF}}_{k}$, such that its state is different from $S_{0}$, and $N_{k}\;$is the number of neighbors with state different from $S_{0}$.

## 4. Hardware Implementation of ALI for Motion Detection

**Figure 5:**
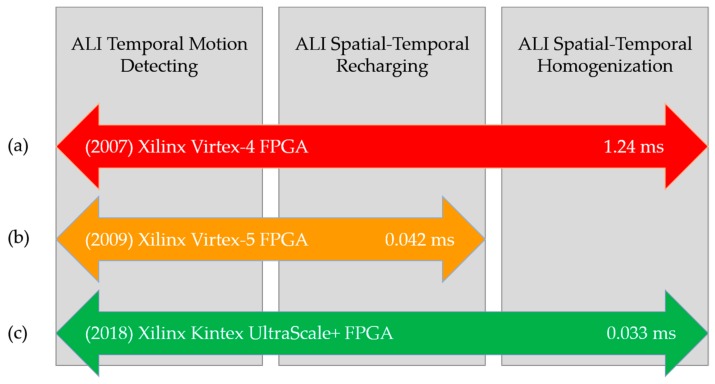
Implementations included in the comparative study. (**a**) ALI implementation with Xilinx Virtex-4 [9]. (**b**) AC implementation with Xilinx Virtex-5 [10]. (**c**) Current ALI implementation with Xilinx Kintex UltraScale+.

In this section, we depict and analyze the current implementation of the ALI algorithm. We also estimate the increase in speed with respect to the previous implementations of the ALI [9] and AC [10] methods, by focusing on the time required by each design to process each video frame. Figure 5 summarizes the characteristics of the implementations considered in this study. Thus, Figure 5a,c offer the processing times for one video frame under an ALI implementation (in years 2007 and 2018, respectively), whereas Figure 5b shows the processing time for an AC implementation (in year 2009).

Again, our current implementation is based on reconfigurable hardware (see Figure 5c). More specifically, we have considered state-of-the-art Xilinx FPGAs [25], and accordingly, we have used the Xilinx Vivado [26] tool for the definition (in VHDL (The VHDL code is available to anyone interested on it for research purposes)), synthesis and implementation of our design. Firstly, we have defined an “ALI module”, which is able to process each image pixel exactly as described in previous sections. Then, starting from this design, we have implemented the complete system, composed of 16 ALI modules and able to process each 4 × 4-pixel image block. The schematics corresponding to both the ALI module and the complete system are provided as Supplementary Material to this paper.

For the implementation, we have considered one of the members of the 16-nm Kintex UltraScale+ family of Xilinx FPGAs. Specifically, we have used a XCKU3P device (the xcku3p-ffvb676-1LV-I model), since the number of IOBs (Input/Output Blocks) provided satisfies the requirements of our design. We have assumed a clock rate of 50 MHz, obtaining the timing parameters relative to maximum delay paths shown in Table 1 after the implementation step.

Therefore, the minimum clock period would be 20.000 − 13.070 = 6.930 ns, that is, the maximum clock rate would be 144.3 MHz. From the previous data, we may also estimate the time required to process each image or frame in a video sequence. Assuming a 320 × 240-pixel image, which is composed of 4800 4 × 4-pixel blocks, and, considering that the maximum data path delay for our implementation is 6.810 ns, the processing would take 0.033 ms at most, which means that the ALI method is capable of processing at least 30 frames per second (fps). Assuming a common video frame rate of 24 fps, we consider that this performance enables real-time sequence processing.

Furthermore, we have compared these timing results with respect to those obtained in our previous FPGA implementation [10], where the AC algorithm was synthetized and implemented in a Xilinx Virtex-5 FPGA (more specifically, the 5vfx30tff665-1 model). For that implementation, which could process 8-pixel image blocks, the maximum combinational data path was 4.348 ns. Therefore, to process a 320 × 240-pixel image, composed of 9600 8-pixel blocks, the previous implementation would require 0.042 ms. This involves an increase in speed of approximately 27% for the current implementation. Similarly, we have computed the performance of the ALI implementation performed in [9] (over a Virtex-4 FPGA), obtaining a frame processing time of 1.24 ms. Note that this is at least two orders of magnitude higher than the performance of the implementation presented in the current work.

Table 2 summarizes the FPGA utilization results for our current implementation. The device utilization rates are very similar to those presented in [10].

Finally, since power consumption is an important issue in current hardware systems, Table 3 summarizes the main results provided by the power analysis performed in our implementation.

## 5. Results

This section includes all the relevant details on the evaluation process carried out to check the performance of the implemented algorithm, which was undertaken using FPGAs to reduce the execution time of the sequential moving object detection algorithm. FPGA data were introduced in the previous section, together with the results of the corresponding analysis.

Three different video sequences were used in this work. These sequences were selected from the ChangeDetection.NET (CDNET) website [27,28]. More concretely, the employed datasets are Corridor, Highway, and wetSnow. The datasets were chosen due to the variable complexity in the motion detection tasks [29,30,31]. In addition, these three benchmarks were chosen to demonstrate that ALI can detect movement in a variety of relatively complex situations.

Firstly, Highway belongs to the 2012 DATASET, and is the simplest dataset of the three that were used. It pertains to the Baseline Category, which represents a mixture of moderate challenges. Some videos have subtle background motion, others have isolated shadows, some have an abandoned object, and others have pedestrians that stop for a short moment and then move away. These videos are fairly easy, but not trivial, to process, and are provided mainly as references. Corridor is a dataset belonging to the 2012 DATASET Thermal Category. The Thermal Category includes videos that have been captured using far-infrared cameras. These videos contain typical thermal artifacts such as heat stamps (e.g., bright spots left on a seat after a person gets up and leaves), heat reflection on floors and windows, and camouflage effects, when a moving object is of the same temperature as the surrounding regions. Lastly, in the 2014 DATSET, we find the Challenging Weather Category that includes outdoor videos captured in challenging winter weather conditions, i.e., snow storm, snow on the ground, fog. We have selected wetSnow, one of the videos belonging to this category.

**Figure 6:**
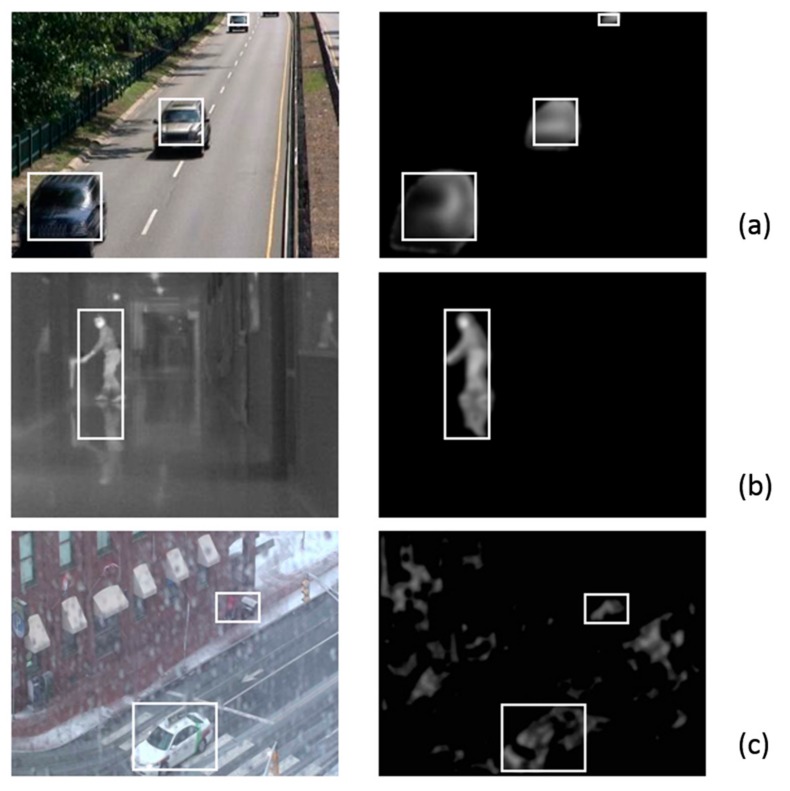
Results of applying the ALI method to three datasets from the ChangeDetection.NET (CDNET) website. (**a**) Highway. (**b**) Corridor. (**c**) wetSnow.

Figure 6 shows the results of applying the ALI method to the three previously described datasets. From top to bottom of the figure, we show the results for Highway (a), Corridor (b) and wetSnow (c). As can be appreciated in the figure, on the right side the results of the ALI method are shown for one of the images from each sequence, along with boxes containing the detected moving objects. On the left of the figure you can see the input image and the boxes surrounding the moving objects. Readers interested in intermediate results of the several phases of the ALI algorithm, presented as images, are invited to consult a paper from the same authors [32]. This paper shows different input image sequences, and their step-by-step outputs, by varying the most important parameters of the ALI algorithm.

ALI behaves excellently when used with the Highway dataset (see Figure 6a). Three cars are perfectly detected and tracked accordingly. A fourth car entering the scene is still not considered, as it is not shown completely. In the case of the Corridor sequence (see Figure 6b), the segmentation by ALI method has also performed in an outstanding manner. In the image presented, a reflection, which is common in thermal images, is included in the surrounding box. Lastly, the most challenging dataset, wetSnow (see Figure 6c), introduces some unwanted movement in several zones of the image.

Table 4 provides more information on the performance metrics of the application of the ALI method to the three datasets. Starting from true positives (TP), false positives (FP) and false negatives (FN), specificity, sensitivity and F-score are shown. These quantitative metrics agree with the qualitative results shown in Figure 6 and the brief explanation provided.

Let us highlight that these performance metrics are in line with previous results of the same authors when applying the ALI method to other datasets, such as MOVI Image Base (http://www.irisa.fr/texmex/ressources/bases/base_images_movi/index.html) [24], Ettlinger-Tor in Karlsruhe (http://i21www.ira.uka.de/image_sequences/) [24,33], TwoWalkNew (University of Maryland) [32,33].

The performance of the ALI method can be broadly compared to other approaches thanks to a recent work [34]. The performance for tasks directly related to motion detection ranges from 24 to 42 fps. More concretely, we have 42 fps for background detection [35], 30 fps for object detection [34], 25 fps for surveillance [36] and video segmentation [37] respectively, and finally, 24 fps for denoising [38]. An objective comparison is quite difficult, as the tasks do not all have the same complexity, and image sizes are also different. However, the previous figures show that our ALI algorithm, implemented in current FPGAs, is competitive for most motion-detection-based computer vision applications.

## 6. Conclusions

In recent years, our research team has been working with the accumulative computation (AC) and algorithmic lateral inhibition (ALI) methods to accurately detect moving objects in video sequences. Moreover, real-time processing of the video images has also been a major issue in all computer vision applications. Unfortunately, the ALI method is computationally intensive, which necessitates maintaining the latest FPGAs to speedup real-time video processing.

To address this problem, the present paper has developed its three main contributions. Firstly, the formal model of finite state machines that simplifies the general neurally-inspired ALI algorithm has been reproduced. This was the first step towards reducing the computation time. Second, the formal model was implemented in up-to-date Xilinx FPGA technology, to continue reducing processing time for the reduced ALI algorithm. Lastly, a comparison between FPGA-based implementations of AC and ALI (about ten years ago) and ALI (to date) has been performed.

We have concluded that the current FPGA-based implementation of ALI achieves excellent performance in terms of F-score (0.98 and 0.86 for simple and complex datasets respectively) as expected, and outperforms the processing times of the AC and ALI implementations performed about ten years ago (27% and 3,658% faster respectively). Current FPGA technology has demonstrated that it is possible to maintain excellent motion detection accuracy whilst implementing more sophisticated biologically-inspired computer vision algorithms.

In the different phases of the ALI algorithm, certain pixel-based processing is performed for each image as it is received, and for the intermediate images generated throughout the processing. In most cases, pixel computation could be performed simultaneously on all pixels, since there is no dependence on such processing. GPUs are well-suited to this parallelism. Therefore, we are planning to translate the ALI algorithm to a GPU-based computing platform. In this way, it will be also possible to compare the current FPGA-based performance with that of a GPU.

## Figures and Tables

**Table 1:** Timing results (summary).

Parameter	Value (in ns)	Description
Slack	13.070	Data required time–Data arrival time (positive values indicate that the path requirement is met)
Requirement	20.000	Clock cycle time (clock period); 50 MHz
Data Path Delay	6.810	Accumulated delay for the worst (slowest) path in the circuit

**Table 2:** Utilization results (summary).

Resource	Used	Available	Utilization (in %)	Description
CLB LUTs	901	162,720	0.55	Logic blocks used as lookup tables (either logic or memory)
CLB Registers	512	325,440	0.16	Logic blocks used as registers (either flip flops or latches)
Bonded IOB	196	280	70	Input/Output ports

**Table 3:** Power results (summary).

Parameter	Value (in W)
Total On-Chip Power	0.476
Dynamic	0.056
Device Static	0.421

**Table 4:** Performance metrics.

Dataset	Frames	TP	FP	FN	Specificity	Sensitivity	F-score
Highway	1700	73,724	556	2520	0.9925	0.9669	0.9795
Corridor	5400	73,263	1882	1882	0.9749	0.9779	0.9764
wetSnow	3500	293,677	9447	85,676	0.9688	0.7741	0.8606
